# Genetic Differentiation and Mixed Reproductive Strategies in the Northern Corn Leaf Blight Pathogen *Setosphaeria turcica* From Sweet Corn in Fujian Province, China

**DOI:** 10.3389/fmicb.2021.632575

**Published:** 2021-05-26

**Authors:** Yuli Dai, Lin Gan, Chengzhong Lan, Xuesong Lu, Xiujuan Yang, Zhimou Gao

**Affiliations:** ^1^Fujian Key Laboratory for Monitoring and Integrated Management of Crop Pests, Institute of Plant Protection, Fujian Academy of Agricultural Sciences, Fuzhou, China; ^2^Department of Plant Pathology, College of Plant Protection, Anhui Agricultural University, Hefei, China

**Keywords:** *Setosphaeria turcica*, northern corn leaf blight, mating type, genetic diversity, population structure, sexual reproduction

## Abstract

The northern corn leaf blight (NCLB) pathogen *Setosphaeria turcica* (Luttrell) Leonard and Suggs is one of the main biotic constraints on sweet corn (*Zea mays* L.) yield and quality in Fujian Province, China. Currently, however, there is comparatively little information available regarding the distribution of mating types, population genetics, and reproductive strategies of this pathogen in Fujian. In this study, we investigated the distribution of mating types and population genetics of 117 isolates of *S. turcica* collected from seven of the main sweet corn-growing regions in Fujian Province, based on multiple polymerase chain reaction analyses using two mating type-specific primer pairs and 11 inter-simple sequence repeat markers. Furthermore, we examined the mode of reproduction of Fujian *S. turcica* populations. Both MAT1-1 and MAT1-2 mating types were detected throughout all seven sampling locations. The majority of MAT1-2 isolates were detected from Dongyou, Jian’ou, Pingnan, Songxi, and Longyan, whereas a large proportion of the detected MAT1-1 isolates were among those collected from Dongfeng and Nanjing. Furthermore, we detected five shared multi-locus haplotypes among *S. turcica* isolates from Dongyou, Jian’ou, Pingnan, Nanjing, and Songxi, whereas no shared haplotypes were observed between the Dongfeng (or Longyan) population and these five populations. Pairwise comparisons of the indices Φ_PT_ and Nm, and population structure and principal coordinate analyses indicated genetic differentiation between both the regional and the mating type populations of *S. turcica* in Fujian. The skewed mating type ratio associated with low a haplotypic diversity and evident linkage disequilibrium reveals a mixed reproductive strategy for *S. turcica* populations in Fujian Province. The findings of this study advance our current understanding of the genetic diversity, population structure, and reproductive strategies of *S. turcica* populations infecting sweet corn in Fujian Province, and will potentially contribute to further resistance breeding efforts.

## Introduction

The heterothallic ascomycetous phytopathogen *Setosphaeria turcica* (Luttrell) Leonard and Suggs) [anamorph, *Exserohilum turcicum* (Pass.) Leonard and Suggs], the causal agent of northern corn leaf blight (NCLB), is a biotic constrain factor, which worldwide is responsible for substantial reductions in the yield and quality of corn (*Zea mays* L.), a vital basic human food source. Moreover, more than half of the global production is used as an industrial raw material and as a source of livestock fodder (Zwonitzer et al., 2009; Nieuwoudt et al., 2018). The symptoms of NCLB initially appear on the basal leaves and subsequently spread to the middle and upper leaves. The typical NCLB symptoms manifest as gray-green, long fusiform, water-soaked lesions on young leaves and later developed into larger necrotic lesions (Vieira et al., 2014; Shi et al., 2017). In susceptible corn cultivars, these necrotic lesions expand and merge, causing extensive damage to the majority of leaves, thereby markedly weakening the photosynthetic capacity of corn plants (Saito et al., 2018; Kotze et al., 2019). Although outbreaks of NCLB occur worldwide, and in susceptible corn cultivars can result in severe yield losses of more than 60%, these outbreaks tend to be most frequent in low latitude regions characterized by temperate and humid conditions (Dingerdissen et al., 1996; Tefferi et al., 1996; Ferguson and Carson, 2004).

The destructive phytopathogenic foliar fungus *S. turcica* is a heterothallic ascomycete that harbors a specific mating type (MAT) gene locus that can occur in two alternative allelic forms, designated MAT1-1 and MAT1-2 (Luttrell, 1958; Gao, 2009). Although the sexual stage for *S. turcica* has rarely been observed on corn leaves in the field, it can frequently be induced under laboratory conditions (Luttrell, 1958; Fan et al., 2007), and the findings of previous studies have indicated that the pathogenicity and parasitic fitness of some sexual progenies of *S. turcica* are markedly enhanced following sexual reproduction (Nelson et al., 1965; Guo et al., 2013). Moreover, some studies have demonstrated that different physiological races of *S. turcica* isolates, characterized by different mating types, can randomly mate and generate diverse novel physiological races with varying degrees of pathogenicity (Moghaddam and Patak, 1994; Hou et al., 2006). Accordingly, these observations indicate that sexual reproduction may be an important source of genetic diversity in *S. turcica*. With numerous new physiological races continually appearing in the main corn-growing countries (Welz et al., 1993; Ferguson and Carson, 2007; Muiru et al., 2010b; Zhang et al., 2011, 2012; Weems and Bradley, 2018; Jindal et al., 2019), an increasing number of studies have focused on investigating the distribution of mating types, genetic diversity, and population structure of *S. turcica* (Borchardt et al., 1998b; Ferguson and Carson, 2004; Haasbroek et al., 2014; Tang et al., 2015; Human et al., 2016; Nieuwoudt et al., 2018; Ma et al., 2020).

To date, several types of molecular marker have been successfully applied in analyzing the genetic diversity and population structure of *S. turcica* populations from Europe (Borchardt et al.,1998a,b; Muiru et al., 2010a), the United States (Ferguson and Carson, 2004, 2007), South Africa (Haasbroek et al., 2014; Human et al., 2016; Nieuwoudt et al., 2018), and northern and southwest China (Fan et al., 2007; Gu et al., 2008; Tang et al., 2015; Li et al., 2019; Ma et al., 2020), including amplified fragment length polymorphism (AFLP), universally primed polymerase chain reaction (UP-PCR), random amplified polymorphic DNA (RAPD), simple sequence repeat (SSR; or microsatellite), sequence-related amplified polymorphism (SRAP), and inter-simple sequence repeat (ISSR) markers. Due to the notable sensitivity, reproducibility, dependability, and preponderance in simultaneous analysis of a large number of loci, ISSR markers are frequently used in population genetic investigations (Kantety et al., 1995; Dai et al., 2020a), and previous genetic analyses using these markers have indicated that *S. turcica* populations from northern China (northeast, north, and northwest) exhibit high within population genetic similarities, but show evident genetic differentiation from *S. turcica* populations in southwestern China (Ma et al., 2020). These observations thus imply that *S. turcica* populations in northern China may be genetically differentiated from those in other corn-growing regions in China (e.g., the south and southeast). However, although natural populations of *S. turcica* in north and southwest China tend to be well characterized with respect to mating type distribution, genetic diversity, population structure, and mode of reproduction (Fan et al., 2007; Guo et al., 2013; Li et al., 2019; Ma et al., 2020), there have been relatively few similar studies on *S. turcica* isolates collected from southeast China, where Fujian Province is one of the most important sweet corn-producing regions in China (Dai et al., 2020a).

Given the high frequency of NCLB outbreaks on sweet corn in Fujian, we sought in the present study to determine the mating type distribution, genetic differentiation, and population structure of *S. turcica* isolates from sweet corn in this province based on multiple PCR analysis using two previously developed mating type-specific primers and ISSR markers (Dai et al., 2019). In addition, we also wished to determine the potential reproductive strategy of *S. turcica* isolates in Fujian. We believe that the findings of this study will enhance our current understanding of the genetic diversity and structure and reproductive strategies of *S. turcica* populations infecting sweet corn in Fujian Province.

## Materials and Methods

### Isolation of *Setosphaeria turcica*

Fujian Province has a subtropical maritime monsoon climate characterized by warmth and humidity and contains three representative sweet corn-growing regions: the east alpine single-cropping sweet corn region, the west-central mountainous double-cropping sweet corn region, and the north and south mountainous triplex-cropping sweet corn regions. In the present study, typical NCLB-symptomatic leaves were randomly collected from the following seven sweet corn-growing regions in Fujian Province: Dongyou (DY; 118°38′15″E, 27°10′44″N), Dongfeng (DF; 118°29′58″E, 27°06′06″N), Jian’ou (JO; 118°18′06″E, 27°01′30″N), and Songxi (SX; 118°47′56″E, 27°32′40″N) regions, which are located in north Fujian; Pingnan (PN; 118°59′58″E, 27°54′27″N), Nanjing (NJ; 117°21′45″E, 24°30′41″N), and Longyan (LY; 117°01′59″E, 24°59′37″N) regions located in east, south and west Fujian, respectively. The straight-line distance between any two adjacent regions is at least 15 km (Supplementary Table 1). Three leaves were randomly collected from each field when sweet corn was approaching the silking stage and at least nine fields were sampled at each location. One isolate was recovered from each leaf through surface sterilizing infected leaf tissues (4 mm × 10 mm) in 75% (v/v) alcohol for 2 min and in 0.1% (w/v) corrosive sublimate water solution for 90 s, with three subsequent washes in sterilized water, and air drying on germ-free cotton gauze. The sterilized tissues were placed on the surface of potato dextrose agar (PDA) (potato 200 g L^–1^, dextrose 20 g L^–1^, agar 18 g L^–1^) and incubated in darkness at 25°C for 3–5 days (Dai et al., 2020a). Thereafter, a 6-mm piece of agar containing fresh tip mycelia from the margin of 5-day-old colonies of each isolate was transferred onto a fresh plate containing 20 mL PDA, and single-spore isolates were obtained as described previously (Dai et al., 2020a). Finally, one rapidly developed well-grown single-spore isolate from each field was randomly selected to give a total of 117 natural isolates from the seven sweet corn-growing regions in Fujian: DY (23 isolates), JO (27), PN (15), NJ (9), SX (18), DF (14), and LY (11). These pure culture isolates were subsequently used for analyses of mating type distribution, genetic diversity, and population structure. In addition, eight heavily infected sweet corn leaves, which were randomly collected from eight fields (one leaf in each field) in NJ (three fields), DF (3), and PN (2) when the corn was nearing maturity, were used to investigate the mating type distribution within single leaves. Pure monosporic isolates were recovered from each lesion and leaf as described above. To identify the fungal isolates, the morphological characteristics of 7-day-old cultures of each isolate were observed by light microscopy (Nexcope NE910, China) under 10 × 40 magnification (Supplementary Figure 1). In addition, each isolate was also identified based on molecular method using two mating type-specific primer pairs as described below. Pure culture isolates were cultivated on pieces of sterile filter paper (1.0 cm × 1.5 cm) on PDA and placed in sterile Kraft paper bags (10 cm × 6 cm) for long-term storage at −20°C.

### DNA Extraction

For the purposes of DNA extractions, we cultured individual *S. turcica* isolates on PDA at 25°C under darkness for 7 days, with each isolate being grown on two 90-mm Petri dishes. For each isolate, we selected a Petri dish showing good mycelial growth for the extraction of DNA using a mini-preparation approach. Mycelia were gently scraped from each Petri dish and placed into individual 2.0-mL microfuge tubes, to which was added an 800-μL volume of extraction buffer [2% CTAB (w/v), 100 mmoL⋅L^–1^ Tris–HCl (pH 8.0), 20 mmoL⋅L^–1^ EDTA, 1.4 moL⋅L^–1^ NaCl, and 10% SDS (w/v)] and an equal volume of phenol/chloroform/isoamyl alcohol (25:24:1). The tube contents were then lightly homogenized and incubated in a 180 rpm shaking incubator at 37°C for 1–1.5 h. Following centrifugation at 12000 rpm for 20 min, the liquid supernatant (approximately 600 μL) from each tube was transferred to a fresh 1.5-mL tube, to which an equal volume of ice-cold isopropanol was added and allowed to precipitate at −20°C for more than 30 min. The resulting DNA preparations were washed one to two times with 70% ice-cold ethanol and resuspended with 50 μL TE buffer (10 mmoL⋅L^–1^ Tris–HCl, 1 mmoL⋅L^–1^ EDTA, pH 8.0). The concentration of each DNA sample was measured using a NanoDrop 2000 spectrophotometer (Thermo Scientific, Shanghai, China) and adjusted to 100 ng⋅μL^–1^ using TE buffer. The DNA samples were then stored in a freezer at −20°C until used for analysis.

### Mating Type Determination

The mating type of isolates was determined based on multiple PCR analyses using two mating type-specific primer pairs (StMAT01-2 and StMAT02-3), the sequences of which are as follows: MAT1-1 (StMAT01-2F: 5′-TGCCTTTGTTGGATTTCG-3′, StMAT01-2R: 5′-CATCGTTCTGGCTGTGGG-3′), with a specific amplicon size of 816 bp; and MAT1-2 (StMAT02-3F: 5′-TACACCAAACAACATCGCTCCT-3′, StMAT02-3R: 5′-TCGGCGTCGTAGAACAAG-3′), with a specific amplicon size of 132 bp (Dai et al., 2020b). PCR was performed using 2× Premix Taq (Takara Biotech Co., Ltd., Dalian, China), with each 25-μL PCR mixture containing 12.5 μL of Premix Taq, 10 pmol of each primer, and 100 ng DNA. Reactions were carried out using a C1000 Touch^TM^ Thermal Cycler (Bio-Rad Laboratories, Life Science Research, Hercules, CA, United States) with initial denaturation at 94°C for 5 min, followed by 35 cycles at 94°C for 45 s, 57.2°C for 45 s, and 72°C for 60 s, and a final cycle at 72°C for 10 min. The amplicons were analyzed by 1.0% agarose gel electrophoresis (Biowest, Nuaillé, France) and stained with ethidium bromide (Shanghai Sangon Biotech, China). Each isolate was independently amplified at least three times, and the sizes of amplicons were estimated using a 2000-bp DNA marker (Takara Biotech Co., Ltd., Dalian, China). The ratios between MAT1-1 and MAT1-2 from each geographical population and combined isolates were subjected to a Pearson Chi-square test using DPS software v7.05 (Hangzhou Reifeng Information Technology Ltd., Hangzhou, China) to evaluate divergence from the expected ratio of 1:1 at the *P* < 0.05 level. Chi-square tests were not performed on populations derived from single leaves owing to the limited population size.

### Genetic Diversity Analysis

Eleven highly polymorphic, sensitive, and repeatable ISSR primers, which were optimized from the ISSR primer database (Biotechnology Laboratory, University of British Columbia, Vancouver, BC, Canada) as described previously (Dai et al., 2019), were used for further population genetic analysis (Supplementary Table 2). Each 25-μL ISSR-PCR amplification mixture contained 12.5 μL of Premix Taq, 10 pmol primer, and 100 ng DNA. ISSR-PCR reactions were carried out in a C1000 Touch^TM^ Thermal Cycler (Bio-Rad Laboratories, Life Science Research, Hercules, CA, United States) with initial denaturation at 94°C for 5 min, followed by 35 cycles at 94°C for 45 s, 48–56°C for 45 s, and 72°C for 1.5 min, and a final cycle at 72°C for 10 min. The products were separated on 1.5% agarose gels containing ethidium bromide (Shanghai Sangon Biotech Co., Ltd., China) and visualized using a Gel Doc^TM^ XR + Imaging System (Bio-Rad Laboratories, Life Science Research, Hercules, CA, United States). DNA fingerprints across the entire population for each ISSR primer on agarose gels were scanned using Image Lab software v5.2 (Bio-Rad Laboratories, Life Science Research, Hercules, CA, United States) and transformed as 0 and 1 (absence and presence, respectively) matrixes.

For the purposes of genetic analysis, *S. turcica* populations from Fujian Province were grouped according to geographical origins and mating types. Genetic diversity parameters, including the percentage of polymorphic loci (P_L_), observed number of alleles (Na), Shannon’s information index (*I*), Nei’s genetic distance, and Nei’s genetic identity, were automatically calculated based on bootstrap analysis with 1,000 replicates using POPGENE v1.32 software (Nei, 1973; Yeh et al., 1999). The number of unique multi-locus haplotypes (N_M_), number of private loci (N_P_), and Nei’s unbiased gene diversity (*H*_U_) were calculated using GenALEx v6.5 based on 999 permutations (Peakall and Smouse, 2012). Haplotype diversity (*H*_*S*_) was calculated as *H*_*S*_ = (–Σ(*P*_*i*_ln(*P*_*i*_)))/ln(n), where *P*_*i*_ is the frequency of the *i*th haplotype in one population and n is the population size (Groth and Roelfs, 1987). The clonal fraction (C_F_) was calculated using the formula C_F_ = 1–[(N_M_)/(subpopulation size)]. All the aforementioned diversity parameters were calculated based on the full data of the products amplified using the 11 ISSR markers.

### Genetic Differentiation Analysis

To assess genetic differentiation (Φ_PT_; a measure of population genetic differentiation that is analogous to *G*_*ST*_ or *F*_*ST*_) for ISSR markers among regional populations, we calculated the pairwise matrix of Φ_PT_ values with corresponding *P* values and gene flow (Nm) among populations of *S. turcica* from the seven sweet corn-growing regions in Fujian, using the full datasets in GenALEx 6.5 (Peakall and Smouse, 2012). Randomization tests were carried out with 999 permutations to obtain *P* values.

Differentiations within and between the MAT1-1 and MAT1-2 populations from different locations were evaluated by pairwise comparisons of Φ_PT_ and Nm values, which were calculated on the basis of the full datasets using GenALEx 6.5 with 999 permutations (Peakall and Smouse, 2012). Genetic distances between the MAT1-1 and MAT1-2 populations from the seven sweet corn-growing regions were used to establish a phylogenetic dendrogram using MEGA X version 10, based on the unweighted pair-group method with arithmetic averages (Kumar et al., 2018).

Analysis of molecular variance (AMOVA) was carried out based on the full datasets in GenAlEx 6.5 using Nei’s genetic distance with 999 permutations (Nei, 1973; Peakall and Smouse, 2012). Independent analyses were performed to assess deviations among isolates from the seven locations and between the two mating types from different regions. Nei’s genetic distance among *S. turcica* isolates, calculated using full datasets, was used for principal coordinate analysis (PCoA), using GenAlEx 6.5 with 999 permutations to identify those coordinates for grouping isolates from the seven locations (Peakall and Smouse, 2012).

### Genetic Structure Analysis

For the purposes of population genetic structure analysis, the full datasets from the 11 ISSR molecular markers were clustered in accordance with the sampling locations of *S. turcica* isolates and mating types. Population genetic structure analysis was performed using STRUCTURE v2.3.4, a clustering program based on a Bayesian model (Pritchard et al., 2000). Two continuous independent tests, with a burn-in of 100000 iterations and 10000 replicates after burn-in, were performed using STRUCTURE v2.3.4 based on the number of populations (K) from 1 to 8 with six replicates per *K* value. The optimal *K* value with the highest probability and lowest standard deviation was obtained following the method described by Evanno et al. (2005) using Structure Harvester^1^ (Earl and Vonholdt, 2012).

### Determination of Reproductive Strategy

To determine the mode of reproduction in *S. turcica* populations from the seven sweet corn-growing regions in Fujian, we calculated two indices, linkage disequilibrium with means of *I*_*A*_ (index of association) and $\bar{r_{d}}$, using the full and clone-corrected (only one representative for each multi-locus haplotype) datasets from the seven regions using MULTILOCUS v1.3b with 1000 randomizations (Agapow and Burt, 2001). For both datasets, mating type ratios were subjected to Pearson Chi-square tests to examine the goodness of fit between the observed and expected (1:1) ratios using DPS software (Hangzhou Reifeng Information Technology Ltd., Hangzhou, China) at the *P* < 0.05 level.

## Results

### Distribution of *Setosphaeria turcica* Mating Types in Fujian

**FIGURE 1:**
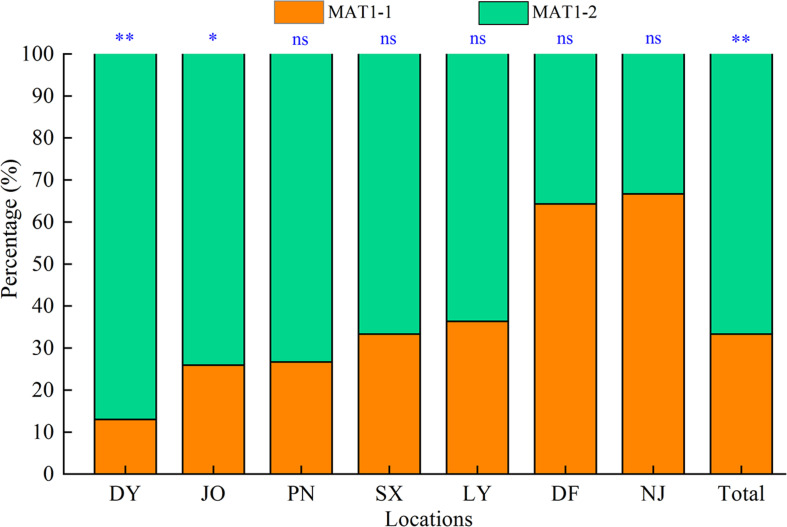
Distribution of the two mating types (MAT1-1 and MAT1-2) of *Setosphaeria turcica* in Fujian Province, China. DY, JO, PN, SX, LY, DF, and NJ represent *S. turcica* isolates collected from the Dongyou (23 isolates), Jian’ou (27), Pingnan (15), Songxi (18), Longyan (11), Dongfeng (14), and Nanjing (9) regions of Fujian Province, respectively. The mating type of 117 *S. turcica* isolates was determined based on multiple PCR analyses using two mating type-specific primer pairs. ^∗^: *P* < 0.05; ^∗∗^: *P* < 0.01; ns: no significant difference.

In this study, we successfully determined the mating types of 117 *S. turcica* isolates obtained from seven locations covering the main sweet corn production regions in Fujian Province based on multiple PCR analyses using two mating type-specific primer pairs, among which, 33.3% (39 isolates) were identified as MAT1-1 and 66.7% (78 isolates) identified as MAT1-2 (Figure 1). Isolates characterized by both mating types were identified in each of the seven sampling locations and we detected a significantly skewed ratio toward the MAT1-2 type within the Fujian population as a whole (Figure 1). More precisely, more than 60% of *S. turcica* isolates from five sampling locations (DY, JO, PN, SX, and LY) were characterized as MAT1-2 (Figure 1), whereas at least 60% of those isolates from DF and NJ were identified as MAT1-1. Furthermore, we identified a skew in the ratios of mating type of *S. turcica* populations from the seven sampling locations, although differences did not reach the level of statistical significance in populations from PN, SX, LY, DF, and NJ (Figure 1). In addition, the results of mating type detection indicated that both MAT1-1 and MAT1-2 can co-occur even on single leaves of diverse cultivars and under different environmental conditions (Table 1). Notably, however, we failed to detect any individual *S. turcica* isolates characterized by both MAT1-1 and MAT1-2 mating types among the assessed field populations, thereby implying that sexual recombination has not occurred in these populations under natural conditions.

**TABLE 1:** The distribution of mating types of *Setosphaeria turcica* within single leaves sampled from three locations (eight fields), identified based on multiple polymerase chain reaction analyses using two mating type-specific primer pairs.

Populations		Years of collection	Locations	Geographical and cropping characterizations	No. of corn cultivars	Mating types		Total
						MAT1-1	MAT1-2	
NJ	Leaf 1	2018	South Fujian	Montanic triplex corn cropping regions	Three	0	4	4
	Leaf 2	2018				4	2	6
	Leaf 3	2019				1	6	7
DF	Leaf 1	2019	North Fujian	Montanic double corn cropping regions	One	1	5	6
	Leaf 2	2020				4	2	6
	Leaf 3	2020				2	6	8
PN	Leaf 1	2016	East Fujian	Montanic single corn cropping regions	Two	1	5	6
	Leaf 2	2020				2	3	5
Total						15	33	48

### Genetic Diversity Among Regional and Between Mating Type Populations

Using 11 ISSR markers, we detected a total of 70 reproducible loci from Fujian *S. turcica* populations, with the number of loci per marker ranging from four (at marker UBC873) to nine (at marker UBC856) (Supplementary Table 2). Of the 70 loci identified for regional populations, the percentage of polymorphic loci ranged from 15.7 to 61.4%, with the lowest and highest percentages being found in the NJ and LY populations, respectively (Table 2). In addition, we detected 75 multi-locus haplotypes in the 117 examined *S. turcica* isolates obtained from seven sweet corn-growing regions, with 60 of these haplotypes being identified only a single time and the remaining 15 being detected at least twice (Supplementary Table 3). Furthermore, five shared multi-locus haplotypes were detected among *S. turcica* isolates from DY, JO, PN, NJ, and SX, whereas we detected no multi-locus haplotypes that were shared between the DF or LY population and any of the other five populations (Supplementary Table 3). Among the 75 multi-locus haplotypes, 18 were observed from JO, whereas only five were observed from NJ (Table 2). The clonal fraction ranged from 0 to 0.44 with the lowest and highest values being obtained for the DF population and the LY and NJ populations, respectively (Table 2). Moreover, we detected significant differences (*P* < 0.05) in the observed number of alleles, Nei’s gene diversity, and Shannon’s information index among the regional *S. turcica* populations (Table 2).

**TABLE 2:** Genetic diversity among regional and between mating type populations of *Setosphaeria turcica* in Fujian Province based on an analysis of inter-simple sequence repeat markers.

Population	*n*	P_L_	N_M_	C_F_	N_P_	*Hs*	Na	*H*_U_	*I*
DY	23	44.3%	13	0.43	0	0.35	1.44 ± 0.50	0.10 ± 0.02	0.16 ± 0.21
JO	27	55.7%	18	0.33	4	0.41	1.56 ± 0.50	0.12 ± 0.02	0.21 ± 0.23
PN	15	28.6%	12	0.20	0	0.30	1.29 ± 0.46	0.10 ± 0.02	0.12 ± 0.20
NJ	9	15.7%	5	0.44	0	0.20	1.16 ± 0.37	0.05 ± 0.01	0.07 ± 0.18
SX	18	48.6%	11	0.39	0	0.30	1.49 ± 0.50	0.09 ± 0.02	0.17 ± 0.21
DF	14	55.7%	14	0	0	0.31	1.56 ± 0.50	0.18 ± 0.02	0.27 ± 0.28
LY	11	61.4%	11	0	8	0.26	1.61 ± 0.49	0.24 ± 0.02	0.33 ± 0.29
Subtotal	117	100%	75	0.36	12		2.00 ± 0.00	0.13 ± 0.01	0.28 ± 0.19
MAT1-1	39	77.1%	28	0.28	0		1.77 ± 0.42	0.18 ± 0.02	0.37 ± 0.26
MAT1-2	78	97.1%	47	0.40	12		1.97 ± 0.17	0.16 ± 0.02	0.42 ± 0.23
Subtotal	117	100%	75	0.36	12		1.97 ± 0.16	0.17 ± 0.01	0.36 ± 0.23

*n: number of isolates; P_*L*_: percentage of polymorphic loci; N_*M*_: number of unique multi-locus haplotypes detected in the population; C_*F*_: clonal fraction, where C_*F*_ = 1–(N_*M*_/n); N_*P*_: number of private loci; *Hs*: haplotype diversity, calculated as *H*_*S*_ = (–Σ(*P*_*i*_ln(*P*_*i*_)))/ln(n), where *P*_*i*_ is the frequency of the *i*th haplotype in one population and *n* is the population size; Na: observed number of alleles; *H*_*U*_: Nei (1987) unbiased average gene diversity; and *I*: Shannon’s information index.*

With respect to populations of the two mating type, we identified a total 54 and 68 polymorphic loci (77.1 and 97.1%, respectively) from MAT1-1 and MAT1-2 populations, respectively (Table 2), and among the 75 multi-locus haplotypes, 28 and up to 47 were observed from MAT1-1 and MAT1-2 populations, respectively (Table 2), with corresponding clonal fractions of 0.28 and 0.40, respectively (Table 2). However, no significant differences between populations of the two mating types were observed with respect to the number of alleles, Nei’s gene diversity, or Shannon’s information index (Table 2).

Six geographical populations of *S. turcica* from DY, JO, PN, NJ, SX, and LY were found to show a high genetic similarity based on the values obtained for Nei’s genetic distance and genetic identity, which were generally smaller than 0.1 and larger than 0.9, respectively (the only exceptions being between populations LY and JO) (Table 3). However, the DF population of *S. turcica* showed a certain extent genetic distance from those the other six regions (Table 3).

**TABLE 3:** Pairwise matrices of Nei’s genetic identity (GI) and Nei’s genetic distance (GD), and gene flow (Nm) and genetic differentiation (Φ_PT_) of regional populations of *Setosphaeria turcica* obtained from seven sweet corn-growing locations in Fujian Province, China.

Population	GI							Nm						
	DY	JO	PN	NJ	SX	DF	LY	DY	JO	PN	NJ	SX	DF	LY
DY (*n* = 23)		0.979	0.963	0.967	0.969	0.881	0.923		4.104	1.877	1.847	2.082	0.679	1.260
JO (*n* = 27)	0.021		0.942	0.952	0.951	0.846	0.886	0.109 (**)		1.258	1.440	1.430	0.564	0.889
PN (*n* = 15)	0.037	0.060		0.986	0.971	0.845	0.911	0.210 (**)	0.284 (**)		7.067	2.348	0.553	1.218
NJ (*n* = 9)	0.034	0.049	0.014		0.971	0.848	0.913	0.213 (0.002)	0.258 (**)	0.066 (0.065)		1.935	0.517	1.201
SX (*n* = 18)	0.032	0.050	0.030	0.029		0.854	0.935	0.194 (**)	0.259 (**)	0.176 (**)	0.205 (**)		0.542	1.565
DF (*n* = 14)	0.127	0.168	0.169	0.165	0.158		0.889	0.424 (**)	0.470 (**)	0.475 (**)	0.492 (**)	0.480 (**)		1.374
LY (*n* = 11)	0.080	0.121	0.094	0.091	0.067	0.118		0.284 (**)	0.360 (**)	0.291 (**)	0.294 (**)	0.242 (**)	0.267 (**)	
	GD							Φ_PT_ with *P* values						

DY, JO, PN, SX, LY, DF, and NJ represent *S. turcica* isolates collected from the Dongyou, Jian’ou, Pingnan, Songxi, Longyan, Dongfeng, and Nanjing regions in Fujian Province, respectively. GI and Nm values are presented above the diagonals in the left and right of the table, respectively. Nm was estimated as Nm = 0.5(1–Φ_*PT*_)/Φ_*PT*_. GD and Φ_*PT*_ with *P* values presented the below diagonals of the left and right panels, respectively. *P* values based on 999 permutations are shown in parentheses below the diagonal of the right panel.**: *P* < 0.001.

### Genetic Differentiation Among Regional and Between Mating Type Populations

With regards to the seven regional populations, we detected a low genetic differentiation and strong gene flow between *S. turcica* populations from PN and NJ (Φ_PT_ = 0.066, *P* = 0.065; Nm = 7.067) and DY and JO (Φ_PT_ = 0.109, *P* < 0.001; Nm = 4.104) (Table 3). However, moderate to high genetic differentiation was detected among populations in the other sampling regions based on pairwise comparisons of the coefficient of Φ_PT_ values with *P* values and Nm values (Table 3).

**FIGURE 2:**
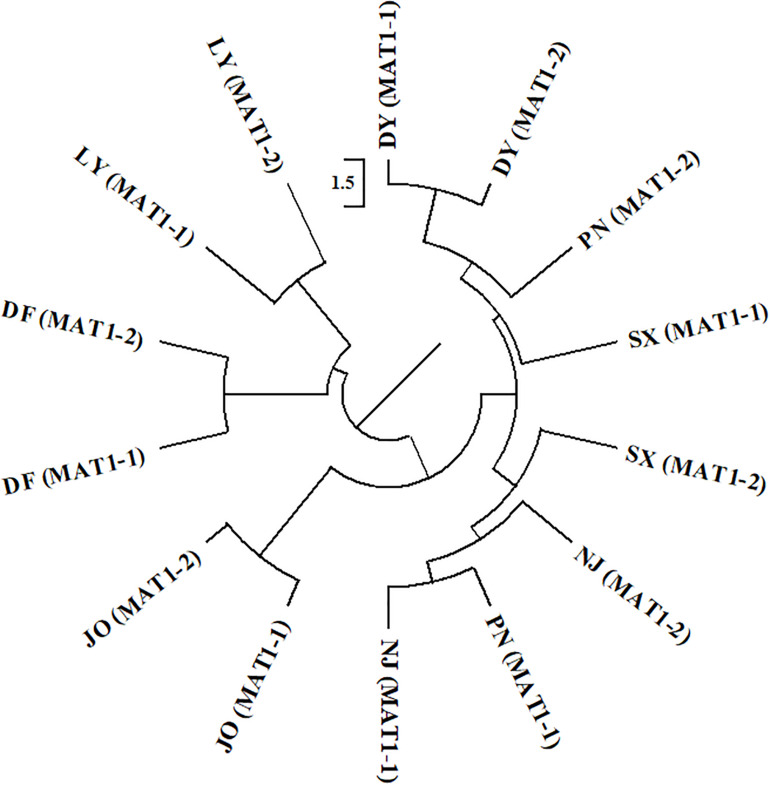
Phylogenetic dendrogram of the two mating types of *Setosphaeria turcica* from seven regions in Fujian Province based of inter-simple sequence repeat markers. DY, JO, PN, SX, LY, DF, and NJ represent *S. turcica* isolates collected from the Dongyou (23 isolates), Jian’ou (27), Pingnan (15), Songxi (18), Longyan (11), Dongfeng (14), and Nanjing (9) regions of Fujian Province, respectively. Isolates of the two mating types obtained from DY, JO, LY, and DF were, respectively clustered, indicating a lack of genetic differentiation between the MAT1-1 and MAT1-2 populations from these regions. The MAT1-1 and MAT1-2 isolates from PN, SX, and NJ were randomly clustered into different groups, implying that there is a genetic differentiation between the two mating type populations from these three regions.

For the two mating type populations, the values of Φ_PT_ ranged from 0.065 (Nm = 7.149) to 0.316 (Nm = 1.082) on the basis of an analysis of regional populations and was 0.018 (Nm = 27.559) on the basis of an analysis of combined isolates (Table 4). Moderate to high genetic differentiation (Φ_PT_ > 0.15) was detected between the two mating type populations in SX, PN, and NJ, whereas we detected low genetic differentiation (Φ_PT_ < 0.15) between these two populations from DY, JO, DF, and LY, respectively (Table 4). Furthermore, phylogenetic analysis indicated that populations of the two mating types from SX, PN, and NJ were randomly clustered into different groups, with different regional sub-populations clustering into the same group (Figure 2), thereby indicating a certain level of genetic differentiation among populations of the two mating types from these three regions. Contrastingly, in the other four populations (DY, JO, DF, and LY), populations of the two mating types from the same region were clustered into the same group (Figure 2), implying no or little genetic differentiation between the two populations in these regions.

**TABLE 4:** Genetic differentiation (Φ_PT_) between the two mating type populations of *Setosphaeria turcica* from seven regions in Fujian Province based on analyses of inter-simple sequence repeat markers.

Parameters	Genetic differentiation between MAT1-1 and MAT1-2 for regional populations							
	DY	JO	PN	NJ	SX	DF	LY	Total
Φ_PT_	0.092	0.065	0.270	0.316	0.194	0.133	0.148	0.018
Nm	4.966	7.194	1.355	1.082	2.077	3.259	2.873	27.559

*DY, JO, PN, SX, LY, DF, and NJ represent *S. turcica* isolates collected from the Dongyou, Jian’ou, Pingnan, Songxi, Longyan, Dongfeng, and Nanjing regions of Fujian Province, respectively. Gene flow (Nm) was estimated as Nm = 0.5(1–Φ_*PT*_)/Φ_*PT*_.*

### Genetic Structure Among Regional and Between Mating Type Populations

**FIGURE 3:**
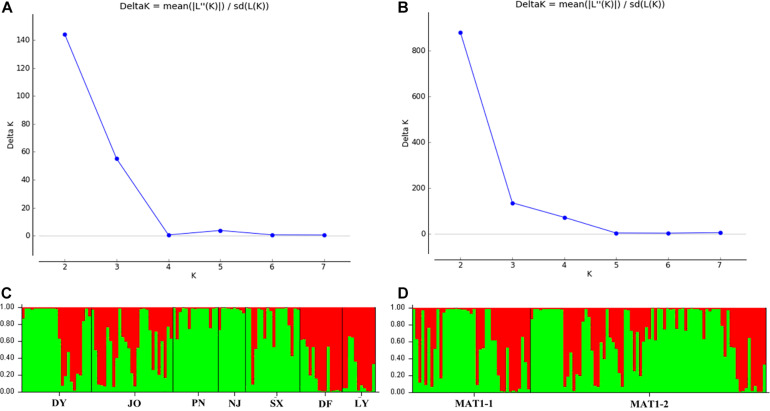
Population structure-inferred membership parameter for 75 multi-locus haplotypes of *Setosphaeria turcica* populations in Fujian Province. Each haplotype is represented by a thin perpendicular bar and the length of each colored bar indicates the membership parameter for each cluster. Individual haplotypes are clustered on the basis of geographical origin **(A,C)** and mating type **(B,D)**, respectively. Sub-figures present the values of Δk and the optimal numbers of *K* = 2 clusters **(A,B)**, and the membership of each haplotype for the two clusters **(C,D)**. Thick black lines divide individual haplotypes into different sub-populations. DY, JO, PN, SX, LY, DF, and NJ represent *S. turcica* isolates collected from the Dongyou, Jian’ou, Pingnan, Songxi, Longyan, Dongfeng, and Nanjing of Fujian Province, respectively.

**FIGURE 4:**
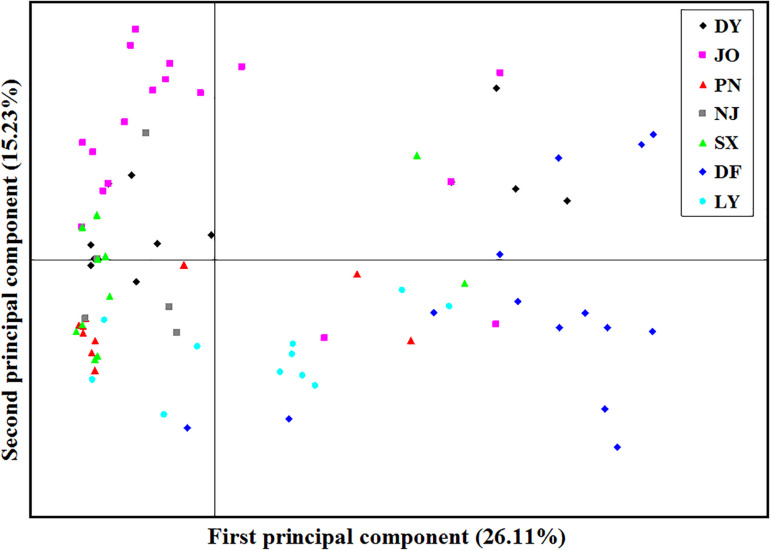
Principal component analysis (PCoA) based on inter-simple sequence repeat marker data for 117 individual isolates collected from seven locations in Fujian Province. Individuals within the same population are marked using the same color and symbol. DY, JO, PN, SX, LY, DF, and NJ represent the *S. turcica* isolates collected from the Dongyou, Jian’ou, Pingnan, Songxi, Longyan, Dongfeng, and Nanjing regions of Fujian Province, respectively. The first and second principal coordinates account for 26.11 and 15.23% of the variation, respectively.

Analysis of the full dataset using STRUCTURE HARVESTER revealed that the optimal number of clusters (highest peak of ΔK appearance) for both the regional and the mating type populations was two (Figures 3A,B). Therefore, for both datasets, the 117 *S. turcica* isolates were divided into two genetic groups (Figures 3C,D). These results indicated no evident correlation between genetic groups and regional (or mating type) populations. A majority of the *S. turcica* isolates could be divided into one or other of the two clusters, with only a small number of isolate admixtures in the two genetic clusters. PCoA analysis indicated two separated clusters, with a majority of the *S. turcica* isolates from different regions plotting to the left of the *y*-axis and the remaining isolates from six locations appearing on the opposite side (Figure 4). The first and second coordinates of the PCoA were found to account for 41.34% of the total variation. Thus, collectively, the results from STRUCTURE and PCoA analyses indicated that *S. turcica* isolates from the seven sweet corn-growing regions in Fujian Province formed two genetically diverged clusters. Furthermore, AMOVA analysis of all seven regional datasets indicated that approximately 69.0 and 31.0% of the total variation occurred within and between populations, respectively (Table 5). In contrast, AMOVA analysis of populations of the two mating types revealed within and between population variations of 99.0 and 1.0%, respectively (Table 5). These results accordingly revealed that the major source of genetic variation in Fujian *S. turcica* populations was derived from within populations. With respect to genetic differentiation among the seven regional populations and between mating type populations, we obtained values of Φ_PT_ = 0.310 (*P* < 0.001) and Φ_PT_ = 0.012 (*P* = 0.105), respectively (Table 5), indicating the occurrence genetic differentiation among the geographical populations of *S. turcica*, although not between populations of the two mating types.

**TABLE 5:** Analysis of molecular variance (AMOVA) among regional populations and between the two mating type populations of *Setosphaeria turcica* in Fujian Province, China, using inter-simple sequence repeat data.

Source	Degree of freedom	Sum of squares	Mean of squares	Estimated variance	Percentage of total variance	Φ_PT_	*P* value
**Among regional populations**							
Among Pops	6	215.904	35.984	1.936	31.0%	0.310	<0.001
Within Pops	110	474.386	4.313	4.313	69.0%		
Total	116	690.291		6.249	100%		
**Between the two mating type populations**							
Among Pops	1	9.521	9.521	0.069	1.0%	0.012	0.105
Within Pops	115	680.769	5.920	5.920	99.0%		
Total	116	690.291		5.989	100%		

### Determination of Reproductive Strategy

An expected ratio of 1:1 between MAT1-1 and MAT1-2 alleles would tend to indicate the likelihood of sexual reproduction within a specific population. In the present study, however, we found that the ratio between MAT1-1 and MAT1-2 for the full and clone-corrected datasets of the DY population was skewed toward MAT1-2 (Table 6). In the other six populations, we did, nevertheless, detect approximately equal mating type ratios for both the full (with the exception of JO) and clone-corrected datasets (*P* > 0.05). For all seven regional populations, there were significant differences in two indices of linkage disequilibrium (*I*_A_ and−$\bar{r_{d}}$) for both the full and clone-corrected (with the exception of NJ) datasets, which is consistent with the hypothesis of non-random mating (Table 6).

**TABLE 6:** Multi-locus linkage disequilibrium analysis of *Setosphaeria turcica* populations collected from seven regions in Fujian Province using full and clone-corrected datasets.

Population	Year of collection	MAT1-1: MAT1-2	*P*-value^*X*^	*I*_A_	−$\bar{r_{d}}$	*P*-value^*Y*^ (*I*_A_ and−$\bar{r_{d}}$)
**The full datasets**						
DY	2018	3:20	<0.001	2.662	0.093	<0.01
JO	2017	7:20	0.021	2.080	0.057	<0.01
PN	2016	4:11	0.121	0.954	0.052	<0.01
NJ	2018	6:3	0.505	1.139	0.115	0.01
SX	2018	6:12	0.239	2.578	0.081	<0.01
DF	2019	9:5	0.423	2.145	0.058	<0.01
LY	2018, 2019	4:7	0.547	1.764	0.042	<0.01
Total		39:78	<0.001	3.031	0.051	<0.01
**Clone-corrected datasets**						
DY	2018	2:10	0.043	1.121	0.039	<0.01
JO	2017	3:11	0.061	1.787	0.048	<0.01
PN	2016	3:7	0.343	0.944	0.056	0.01
NJ	2018	3:2	1.000	1.150	0.128	0.07
SX	2018	4:5	1.000	1.268	0.041	0.01
DF	2019	9:5	0.423	2.145	0.058	<0.01
LY	2018, 2019	4:7	0.547	1.764	0.042	<0.01
Total		28:47	0.038	2.152	0.035	<0.01

*^*X*^*P*-values were calculated by analysis of mating type ratios with Pearson Chi-square tests using DPS software v7.05 (Hangzhou Reifeng Information Technology Ltd., Hangzhou, China). *P* < 0.05 indicates a skewed mating type ratio and an absence of random mating within populations. ^*Y*^Values of *I*_*A*_ (index of association) and−$\bar{r_{d}}$ (multi-locus linkage disequilibrium) were calculated based on an analysis of the ISSR markers data with 1000 randomizations using MULTILOCUS v1.3b (Agapow and Burt, 2001).−$\bar{r_{d}}$ values that deviate markedly (*P* < 0.05) from 0 indicate a clear linkage disequilibrium and support non-random mating.*

## Discussion

In this study, using a multiple PCR with mating type-specific primer pairs and 11 ISSR markers, we investigated the distribution of mating types, population genetic diversity and differentiation, population genetic structure, and potential reproductive strategy of *S. turcica* populations sampled from seven locations covering the main sweet corn-producing regions in Fujian Province, In all seven populations, we identified both of the two mating types, including the co-occurrence of MAT1-1 and MAT1-2 genotypes within eight specifically collected single leaves, despite the unequal distribution of the two mating types in all *S. turcica* populations, thereby implying the widespread co-occurrence of MAT1-1 and MAT1-2 under natural conditions in Fujian. Interestingly, in most sampling locations (DY, JO, PN, SX, and LY; Figure 1), we found that MAT1-1 and MAT1-2 showed evident site-specific diversities and an imbalanced distribution toward MAT1-2, indicating that isolates having a MAT1-2 mating type are perhaps at a greater advantage under the environmental conditions characterizing Fujian. Particularly noteworthy in this regard is that coexistence of *S. turcica* isolates with different mating types within a single leaf was observed within eight fields at three separate sampling locations in Fujian, as this status would be assumed to markedly heighten the potential for future random mating. Nevertheless, in this respect, it should be emphasized that sexual reproduction is not only dependent on the presence of MAT1-1 and MAT1-2 loci but also the compatibility among isolates, adequate nutritive materials, and other suitable conditions (Linde et al., 2003; Dai et al., 2020a). Accordingly, we believe that it would be prudent to initiate a program to monitor the distribution of *S. turcica* mating types in Fujian Province, with the aim of identifying any shifts in mating type distribution, as such shifts are typically accompanied by changes in reproductive strategy.

Gaining an understanding of the mating type distribution of a certain phytopathogens is vital with respect to deducing the level of population genetic variability attributable to the underlying sexual recombination (Valent et al., 1991). In this regard, Gu et al. (2008) revealed that a high level of population genetic variability in *S. turcica* from northern China was notably associated with the distribution of mating types. Our findings for *S. turcica* populations in Fujian Province indicated low to moderate genetic diversities and corresponding DNA polymorphisms among regional populations. The low genetic diversity detected in regional populations can be assumed to reflect the genetic similarity among these populations (Onaga et al., 2015), and with the exception of DNA polymorphisms, similar results were obtained for populations of the two mating types.

For most of the *S. turcica* collection sites in Fujian Province, we detected shared multi-locus haplotypes among isolates (Supplementary Table 3), thereby implying that either large-scale population migration has occurred or that the pathogen been spread via natural agents such as wind or through the long-distance transportation of fresh corn materials (Robert and Findley, 1952; Human et al., 2016), particularly fresh corn stalks, which are a frequently used to prepare silage for livestock. A multi-locus haplotype that was shared between one *S. turcica* isolate from a site in NJ and those from locations in JO, PN, DY, and SX, separated by respective distances of more than 290, 310, 320, and 360 km provides compelling evidence for the large-scale spread of *S. turcica*. Consistently, we also identified a potentially high level of gene flow (Nm = 7.067) between isolates from NJ and those from PN. This evidence for widespread gene drift between the NJ and PN populations of *S. turcica* also tends to be indicative ongoing frequent contact between the two populations, although as previously mentioned, alternative explanations are also possible.

Values obtained for genetic differentiation (Φ_PT_) among the Fujian regional populations ranged from 0.066 to 0.492 (Table 3), indicating that that 50.8 to 93.4% of genetic variation is attributable to that within populations. We also obtained relatively low Shannon information indices (0.07 to 0.33) (Table 2), with an index value of 0.33 indicating that more than 67% of the genetic variability among populations was accounted for by the diversity among individuals. These findings are consistent with those reported by Ma et al. (2020), who obtained Shannon information indices ranging from 0.20 to 0.37, indicating low variation among populations in southwest and north China, although they contrast with the results reported by Human et al. (2016) and Nieuwoudt et al. (2018), who obtained Shannon diversity indices of 0.87 to 0.90 and 0.66 to 0.94, indicating high levels of diversity among South African populations of *S. turcica* from maize and sorghum. We speculate that the low genetic diversity detected among Fujian *S. turcica* populations could be attributed to the fact populations in this province have become established relatively recently, and to date there have been only a limited number of generations.

The influences of geographical location and mating type on population genetic differentiation were examined based on AMOVA and pairwise genetic differentiation between mating types from the different locations. We accordingly found that the within-population variation was lower when *S. turcica* isolates were grouped on the basis of collection region, than when grouped according to mating type. In addition, we detected significant genetic differentiation among isolates from different sampling locations, which was higher than when isolates were grouped according to mating type. These results thus tend to indicate that Fujian *S. turcica* isolates with the same mating type are more similar than isolates from the same location. The results also indicate that mating type has an influence on the genetic differentiation among isolates from the same location. Results obtained from hierarchical analysis of AMOVA were found to contrast with those reported in a previous study, in which the authors examined mating type differentiation of *S. turcica* in Heilongjiang Province, China, and found that within-population variation was higher when isolates were grouped according to geographic location than when grouped based on mating type (93.4 and 85.3%, respectively) (Li et al., 2019). Previous analyses using microsatellite markers have revealed no significant differentiation in *S. turcica* population from the KwaZulu-Natal region in South Africa (Human et al., 2016), whereas Borchardt et al. (1998a, b) observed a regional sub-division of *S. turcica* populations only between those populations separated by large-scale geographical distance. Nevertheless, genetic analyses have revealed genetic difference among South African *S. turcica* populations from corn and sorghum (Nieuwoudt et al., 2018). Our findings tend to be consistent with the conclusions drawn by Borchardt et al. (1998a); Ferguson and Carson (2004), and Ma et al. (2020) that genetically differed among regional *S. turcica* populations from tropical and temperate climates, the eastern United States and China, respectively.

In the present study, we used several approaches to investigate the potential reproductive strategies adopted by *S. turcica* populations infecting sweet corn in Fujian Province. The values we obtained for the haplotypic diversity of *S. turcica* isolates (0.20–0.41) differ from those of the high-haplotypic diversity populations from tropical climates characterized by frequent sexual reproduction (Borchardt et al., 1998a; Ferguson and Carson, 2007). Using both the full and clone-corrected datasets, the values obtained for the MAT1-1 to MAT1-2 ratios of *S. turcica* isolates from PN, NJ, SX, DF, and LY provide evidence of random mating interactions, with only JO isolates showing no significant difference from the expected 1:1 ratio using the clone-corrected dataset. In the case of both complete and clone-corrected datasets, we found that the DY population was skewed toward a MAT1-2 mating type and that the mating type ratio differed significantly from the expected ratio of 1:1. Furthermore, for both full and clone-corrected (with the exception of NJ) datasets, we detected significant differences in linkage disequilibrium (*I*_*A*_ and−$\bar{r_{d}}$) (*P* < 0.05), and thus obtained no convincing evidence in support of the occurrence of random mating in Fujian *S. turcica* populations. Collectively, the low haplotypic diversities, skewed mating type distribution, and conspicuous linkage disequilibrium (*I*_*A*_ and−$\bar{r_{d}}$) tend to be indicative of a clonal or mixed reproductive strategy within the *S. turcica* populations distributed throughout Fujian, which is consistent with finding reported by Human et al. (2016). In addition, although we successfully identified the mating types of 117 field *S. turcica* isolates along with those of a further 48 single leaf isolates based on multiple PCR analyses using two mating type-specific primer pairs, we failed to detect any *S. turcica* isolate with both MAT1-1 and MAT1-2 mating types in these populations. Moreover, our observation of uneven MAT1-1 and MAT1-2 distributions in all examined geographical populations is inconsistent with the hypothesis of frequency-dependent selection (Milgroom, 1996; May et al., 1999), thereby implying that sexual reproduction probably does not occur under natural conditions in Fujian. In order to confirm this assumption, however, it will be necessary to sample at larger spatial and temporal scales in further investigations.

## Conclusion

In conclusion, we detected genetic differentiation among *S. turcica* populations sampled from seven locations in Fujian Province. Our results revealing a skewed mating type distribution, low haplotypic diversities, and significant linkage disequilibrium are indicative of a mixed reproductive strategy among the *S. turcica* populations. Further studies are necessary to clarify the influence of corn varieties on the selection of this pathogen, given the important role played by host resistance in pathogen population selection (Milgroom, 2015). Furthermore, studies using a series of corn lines carrying single resistance genes for physiological race identification would provide further evidence of pathogenic diversity, as currently little is known regarding the physiological races of *S. turcica* or their distribution in Fujian Province.

## Data Availability Statement

The original contributions presented in the study are included in the article/Supplementary Material, further inquiries can be directed to the corresponding author/s.

## Author Contributions

YD, XY, and ZG: design the experiment and further review and editing. YD, LG, and CL: methodology and technology and investigation and data analysis. YD, LG, CL, XL, and XY: samples and isolates collection. YD: writing the draft manuscript. YD and XY: project supporting. All authors contributed to the article and approved the submitted version.

## Conflict of Interest

The authors declare that the research was conducted in the absence of any commercial or financial relationships that could be construed as a potential conflict of interest.

## Footnotes

1 http://taylor0.biology.ucla.edu/structureHarvester/
