# Supplementary material for: Genetic Differentiation and Mixed Reproductive Strategies in the Northern Corn Leaf Blight Pathogen Setosphaeria turcica From Sweet Corn in Fujian Province, China
Source: Front Microbiol. 2021 May 26;12:632575. doi: 10.3389/fmicb.2021.632575 (PMC8187859; doi:10.3389/fmicb.2021.632575)
Supplement: Supplementary file 1 [file Data_Sheet_1.PDF]

Supplementary Information

Supplementary Table S1. Geographical distances among sampling locations and information of sampling sites in Fujian Province, China.

Population	Straight-line distances among sampling locations (km)							Location	Disease severity	Sweet corn cultivars
	DY	DF	JO	SX	PN	LY	NJ			
DY	0							North Fujian	9	Taitian 88, Shangpin, Taixiantian 1, Meiyu 8
DF	16.2	0							5	Taixiantian 1
JO	36.6	24.4	0						3	Taitian 88, Taixiantian 1
SX	43.1	55.0	73.4	0					1	Unknown
PN	44.4	52.5	68.8	71.8	0			East Fujian	7	Shangpin, Super sweet 308, Yuetian 22
LY	290.4	277.3	259.8	332.3	289.7	0		West Fujian	9	Unknown
NJ	323.5	312.0	297.2	366.5	314.4	64.7	0	South Fujian	9	Shangpin, Yongzhen 7, Mintian 6855

DY, DF, JO, SX, PN, LY, and NJ represent *S. turcica* isolates collected from the Dongyou, Dongfeng, Jian’ou, Songxi, Pingnan, Longyan, and Nanjing regions in Fujian Province, respectively. Disease severities of sampling fields were evaluated using a 1 to 9 scale: 1 = no more than 5% of the leaf surface diseased, 3 = 6 to 10% of the leaf surface diseased, 5 = 11 to 30% of the leaf surface diseased, 7 = 31 to 70% of the leaf surface diseased, and 9 = over 70% of the leaf surface diseased.

Supplementary Table S2. Characterization of inter-simple sequence repeat (ISSR) markers used to evaluate the genetic diversity and population structure of *Setosphaeria turcica* in Fujian Province, China.

Marker names	Repeat motifs (5′→3′)	Tm (℃)	N	N_L	*H*t
UBC117	$(AGA)_7$	55.6	7	7	0.257±0.032
UBC118	$(GTC)_6$	53.1	6	6	0.106±0.025
UBC808	$(AG)_8C$	51.2	5	5	0.108±0.004
UBC817	$(CA)_8A$	55.6	6	6	0.194±0.010
UBC847	$(CA)_8RT$	56.0	6	6	0.168±0.031
UBC855	$(AC)_8YT$	53.1	6	6	0.205±0.007
UBC856	$(AC)_8YA$	54.7	9	9	0.062±0.003
UBC857	$(AC)_8YG$	51.2	8	8	0.149±0.028
UBC866	$(CTC)_6$	51.2	6	6	0.345±0.013
UBC873	$(GACA)_4$	48.0	4	4	0.199±0.044
UBC887	$DVD(TC)_7$	51.2	7	7	0.129±0.010
Total	…	…	70	70	…

R: A+G; Y: C+T; D: A+T+G; V: A+C+G; Tm: optimal annealing temperature for each marker; N: number of loci; N_L: number of polymorphic loci; and *H*t: total gene diversity (mean ± standard error) for each markers.

Supplementary Table S3. Sorted multi-locus haplotypes, with repeated matching haplotypes listed first.

Samples	Pops	Genotypes	No.	Label	Shared [X]
DY20	1	000000000000001111100000000000111100001100110000001111111111111001100g	2	A	No
DY17	1	000000000000001111100000000000111100001100110000001111111111111001100g	0	A	
JO08	2	000000000000111111100000000000111100001100110000001111111111111001100g	2	B	Yes
DY19	1	000000000000111111100000000000111100001100110000001111111111111001100g	0	B	
NJ08	4	111000000011111111100000000000111100001100110000111111111111111001111g	3	C	No
NJ07	4	111000000011111111100000000000111100001100110000111111111111111001111g	0	C	
NJ05	4	111000000011111111100000000000111100001100110000111111111111111001111g	0	C	
SX13	5	111111000011111110000000000000111100001100110000001111111111111001111g	4	D	No
SX12	5	111111000011111110000000000000111100001100110000001111111111111001111g	0	D	
SX10	5	111111000011111110000000000000111100001100110000001111111111111001111g	0	D	
SX02	5	111111000011111110000000000000111100001100110000001111111111111001111g	0	D	
SX06	5	111111000011111110000000000000111100001100110000111001111111111001111g	2	E	No
SX05	5	111111000011111110000000000000111100001100110000111001111111111001111g	0	E	
SX17	5	111111000011111110000000000000111100001100110000111111111111111001111g	4	F	No
SX14	5	111111000011111110000000000000111100001100110000111111111111111001111g	0	F	
SX11	5	111111000011111110000000000000111100001100110000111111111111111001111g	0	F	
SX08	5	111111000011111110000000000000111100001100110000111111111111111001111g	0	F	
PN15	3	111111000011111111100000000000000000001100110000111111111111111001111g	2	G	No
PN04	3	111111000011111111100000000000000000001100110000111111111111111001111g	0	G	
JO19	2	111111000011111111100000000000111100001100110000001100111111111001100g	4	H	No
JO17	2	111111000011111111100000000000111100001100110000001100111111111001100g	0	H	
JO12	2	111111000011111111100000000000111100001100110000001100111111111001100g	0	H	
JO09	2	111111000011111111100000000000111100001100110000001100111111111001100g	0	H	
SX07	5	111111000011111111100000000000111100001100110000001100111111111001111g	4	I	Yes
PN09	3	111111000011111111100000000000111100001100110000001100111111111001111g	0	I	
JO18	2	111111000011111111100000000000111100001100110000001100111111111001111g	0	I	
JO10	2	111111000011111111100000000000111100001100110000001100111111111001111g	0	I	
JO23	2	111111000011111111100000000000111100001100110000001111111110000001100g	3	J	No
JO21	2	111111000011111111100000000000111100001100110000001111111110000001100g	0	J	
JO20	2	111111000011111111100000000000111100001100110000001111111110000001100g	0	J	
JO22	2	111111000011111111100000000000111100001100110000001111111111111001100g	4	K	Yes
JO02	2	111111000011111111100000000000111100001100110000001111111111111001100g	0	K	
JO01	2	111111000011111111100000000000111100001100110000001111111111111001100g	0	K	
DY05	1	111111000011111111100000000000111100001100110000001111111111111001100g	0	K	
SX01	5	111111000011111111100000000000111100001100110000001111111111111001111g	14	L	Yes
NJ04	4	111111000011111111100000000000111100001100110000001111111111111001111g	0	L	
PN14	3	111111000011111111100000000000111100001100110000001111111111111001111g	0	L	
JO24	2	111111000011111111100000000000111100001100110000001111111111111001111g	0	L	
JO06	2	111111000011111111100000000000111100001100110000001111111111111001111g	0	L	
DY22	1	111111000011111111100000000000111100001100110000001111111111111001111g	0	L	
DY12	1	111111000011111111100000000000111100001100110000001111111111111001111g	0	L	
DY10	1	111111000011111111100000000000111100001100110000001111111111111001111g	0	L	
DY09	1	111111000011111111100000000000111100001100110000001111111111111001111g	0	L	

DY08	1	111111000011111111110000000000111100001100110000001111111111111001111g	0	L	
DY07	1	111111000011111111110000000000111100001100110000001111111111111001111g	0	L	
DY06	1	111111000011111111110000000000111100001100110000001111111111111001111g	0	L	
DY04	1	111111000011111111110000000000111100001100110000001111111111111001111g	0	L	
DY03	1	111111000011111111110000000000111100001100110000001111111111111001111g	0	L	
NJ06	4	111111000011111111110000000000111100001100110000111111111111111001111g	5	M	**Yes**
NJ03	4	111111000011111111110000000000111100001100110000111111111111111001111g	0	M	
NJ02	4	111111000011111111110000000000111100001100110000111111111111111001111g	0	M	
PN10	3	111111000011111111110000000000111100001100110000111111111111111001111g	0	M	
PN01	3	111111000011111111110000000000111100001100110000111111111111111001111g	0	M	
PN12	3	111111000011111111110000000000111100111100110000111111111111111001111g	2	N	No
PN11	3	111111000011111111110000000000111100111100110000111111111111111001111g	0	N	
DY02	1	111111000011111111110000000000111111001100110000001111111111111001111g	2	O	No
DY01	1	111111000011111111110000000000111111001100110000001111111111111001111g	0	O	
DF04	6	000000000000001111000000000000001111000011001100000011001100000000000011g	1	1	No
DF01	6	000000000000001111000000000000001111000011001100000011111111111111001111g	1	2	
DF07	6	000000000000001111000000000000001111000011001100110011111111111111001111g	1	3	
DF09	6	000000000000001111000000000000001111000011111111111111111111110000001111g	1	4	
DF06	6	000000000000001111000000000001111110000111111111111111111111111001111g	1	5	
DY15	1	000000000000001111110000000000001111000011111100000000111111111111001100g	1	6	
DF14	6	000000000000001111110000000000001111000011111100110000111111110000001100g	1	7	
DF11	6	000000000000001111110000000000001111000011111100110011111111110000001100g	1	8	
DF12	6	000000000000001111110000000000001111000011111100110011111111111111001111g	1	9	
DF13	6	000000000000001111110000000000001111000011111111100111111111111111001111g	1	10	
DF03	6	000000000000001111110000000001111110000110011000000110011111111111001111g	1	11	
DF08	6	000000000000001111110000000001111110000111111111111111111111111111001111g	1	12	
LY07	7	000000000000111111000000000000001111000000000000000000111111111111111001111g	1	13	
SX03	5	000000000000111111000000000000001111000011001100000000111111111111001111g	1	14	
JO05	2	000000000000111111110000000000001111000011001100000011111111110000000000g	1	15	
DY18	1	000000000000111111110000000000001111000011001100000011111111110000001100g	1	16	
PN06	3	000000000011001111110000000000000000000011001100001111111111111111001111g	1	17	
PN13	3	000000000011111111110000000000000000000011001100000011111111111111001111g	1	18	
SX16	5	000000000011111111110000000000001111000011001100000000111111110000001111g	1	19	
JO27	2	000000001100001111110000000000001111000011001100001111111111111111001111g	1	20	
LY09	7	001100000011111111000000000000001111000011111100110000111111110011001111g	1	21	
JO15	2	001111000011111111110000000000001111000000001111000011001111110000001100g	1	22	
JO14	2	001111000011111111110000000000001111000011001100000011001111110011001100g	1	23	
NJ09	4	001111000011111111110000000000001111000011001100001111111111111111001111g	1	24	
DF02	6	110000000000001111000000000000001111000011001100000011111111111111001111g	1	25	
LY01	7	110000000011111111000000000001111110000000000000000001111111111111111111g	1	26	
LY08	7	111100000011110011000000000000001111000011111111100111111111111111001111g	1	27	
DY14	1	111100000011111100000000000000001111000011001100000011111111111111001100g	1	28	
LY05	7	111100000011111111000000000000001111000011111100110011111111111111111111g	1	29	
LY06	7	111100000011111111000000000000001111000011111111111111111111110011001111g	1	30	
PN03	3	111100000011111111110000000011111100001100110000001100111111111001111g	1	31	
DF05	6	111100000011111111110000000011111100001111111111111111111111111001111g	1	32	

LY11	7	1111000000111111111111001100001111000011111111110011111111111111001111g	1	33
JO26	2	1111001100000011111100000000000111100001100110000111111111111111001111g	1	34
DY21	1	1111111000011110000110000000000111100001100110000001111111111111001111g	1	35
JO25	2	1111111000011110011000000000000111100001100110000001100111111111001100g	1	36
DY13	1	1111111000011111100000000000000111100001111110000001111111111111001100g	1	37
PN05	3	1111111000011111111000000000000000000001100110000111111111111111001111g	1	38
LY02	7	1111111000011111111000000000000111100000000000000001111111111111111111g	1	39
LY03	7	1111111000011111111000000000000111100000000000000111111111111111111111g	1	40
SX09	5	1111111000011111111000000000000111100000000110000111111111111111001111g	1	41
SX15	5	1111111000011111111000000000000111100001100110000000000000111111001111g	1	42
DF10	6	1111111000011111111000000000011111100001111110011111111111111111001111g	1	43
PN07	3	1111111000011111111110000000000110000001100110000111111111111111001111g	1	44
SX04	5	1111111000011111111110000000000111100001100110000000000111100111001111g	1	45
JO03	2	1111111000011111111110000000000111100001100110000000011111110000000000g	1	46
JO04	2	1111111000011111111110000000000111100001100110000000011111110000001100g	1	47
NJ01	4	1111111000011111111110000000000111100001100110000111111111110000000000g	1	48
DY16	1	1111111000011111111110000000000111100001111110000001100111111111001100g	1	49
DY23	1	1111111000011111111110000000000111100001111110000001111111111111001111g	1	50
DY11	1	1111111000011111111110000000000111100111100110000001111111111111001111g	1	51
JO16	2	1111111000011111111110000000000111110000001100000011001111111111001100g	1	52
JO11	2	1111111000011111111110000000000111110011001100000011001111110000000000g	1	53
JO13	2	1111111000011111111110000000000111110011001100000011001111110000001100g	1	54
JO07	2	1111111000011111111110000000000111110011001100000011111111110011001100g	1	55
PN02	3	1111111000011111111110000000001111110000000000000001111111111111001111g	1	56
PN08	3	1111111000011111111110000000001111110000110011000011111111111111001111g	1	57
SX18	5	1111111000011111111110000001100111100111100110000111111111111111001111g	1	58
LY04	7	1111111000011111111110000001111111100111111110011111111111111111111111g	1	59
LY10	7	1111111000011111111111111111111111100001111110011000011001100111111111g	1	60

^{X} One haplotype was shared by single isolates from different locations.

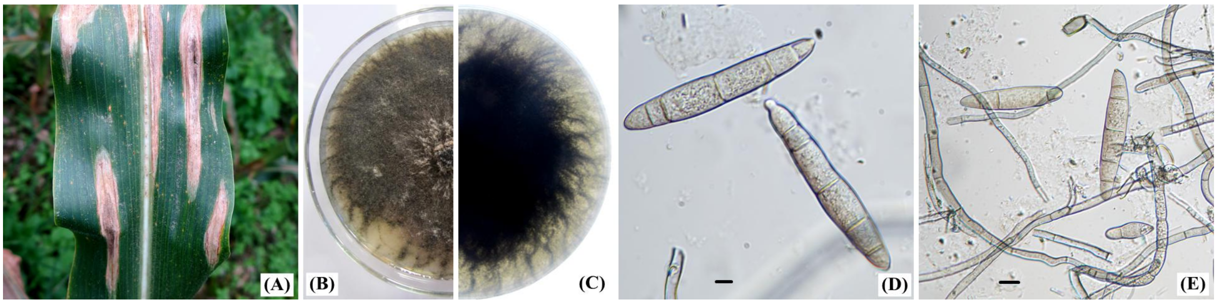

Supplementary Figure S1. Symptoms of northern corn leaf blight on naturally infected corn leaf and morphological characteristics of *Setosphaeria turcica*.

(A): Symptoms of natural infection with northern corn leaf blight on corn leaf. (B)–(C): Top (B) and reverse (C) view of colony characteristics of the fungal pathogen on potato dextrose agar media seven days post inoculation. (D): Mature conidia. (E): Hyphae and conidia. Bars = 10 μm.
